# Cerebral Compression1Read at the one hundred and fourth annual meeting of the Medical Society of the State of New York, held at Albany, April 18 and 19, 1911.

**Published:** 1911-07

**Authors:** Edgar R. McGuire

**Affiliations:** Attending Surgeon, Buffalo General Hospital; Clinical Professor of Surgery, Medical Department, University of Buffalo


### Cerebral Compression[1]

By EDGAR R. McGUIRE, M.D.
Attending Surgeon, Buffalo General Hospital
Clinical Professor of Surgery, Medical Department, University of Buffalo

ANATOMISTS describe three distinct membranes covering the brain; but from the clinical aspect there are only two: the dura and the pia-arachnoid, which are so closely associated clinically that we can speak of them as one. The brain is sustained inside the skull in such a way as to be practically bathed in fluid. This is equally true of the spinal cord; and we must bear in mind the communication between the fluid around the spinal cord and that around the brain through the foramen of Magendie, located at the floor of the fourth ventricle. The circulation of this fluid throughout the brain is of importance. The choroid plexuses, while projecting into the ventricles, are really separate and distinct from them. The fluid is secreted by the choroid plexuses into the ventricles; thence it passes into the subarachnoid space through various openings. (Monroe, Magendie, and Luschka.) Here it is taken up by the Pacchionian bodies and the cerebral veins. One can readily see the importance of a clear understanding of this circulation; since any obstruction to this outflow will become an important factor in producing cerebral compression.

The lymphatics of the brain have not been studied as carefully as they should have been. It is usually assumed that in an

---

1. Read at the one hundred and fourth annual meeting of the Medical Society of the State of New York, held at Albany, April 18 and 19, 1911.

organ freely drained by lymphatics, an infection of that organ will produce marked constitutional symptoms. This is very well illustrated in infections of the gall-bladder and ducts, as we know that lesions confined to the gall-bladder produce a minimum of effect compared with the infections of the common duct, the latter being freely drained by the lymphatics. This point may well account for the clinical fact which has long been known—namely, that in diseases of the brain when a rise of temperature occurs probably either an involvement of the meninges or a thrombophlebitis is present and, therefor, the case has a much graver aspect than such symptoms would indicate in many other lesions. In one instance I have seen the whole frontal lobe involved in a large abscess without elevation of temperature. One can scarcely imagine this condition present in any other part of the body without producing more marked symptoms.

The arterial circulation of the brain is carried on by means of the carotids and vertebrals. The venous circulation, however, is exceedingly intricate. Generally speaking the veins empty into the sinuses, and these into the jugulars. Again one may see how important the venous circulation becomes, since any obstruction to its outflow produces edema, and this is one of the prominent factors in cerebral compression.

Consideration of brain lesions may be divided under the following heads: first, contusion; second, concussion; third, compression.

It is exceedingly important to appreciate definitely the pathology of each of these lesions, because the treatment will depend entirely upon which type be present. In contusion and concussion of the brain we expect recovery to occur without any surgical interference; but when we encounter the other lesions the only hope of relief lies in surgery.

There has been some confusion regarding the terms contusion and concussion, even to the extent of eliminating at least one of them. Under both of these heads one should include minute hemorrhages or edema, producing unconsciousness from a few seconds up to ten or twelve days. These are known to be present even though they are very difficult to demonstrate even microscopically.

Compression, however, has a very definite meaning. Under this head we must include any lesions whereby pressure is exerted upon the brain. These vary in frequency in about this order: first, hemorrhage; second, depression of bone due to injury of skull; third, excess of fluid; fourth, infections of the membrane; fifth, tumors; sixth, bullets or other foreign bodies; seventh, pachy- and lepto-meningitis.

For our purpose it will be necessary to consider only a few of these conditions as the majority of them are thoroughly understood.

Those cases coming under the head of excessive fluid may be of several varieties. In fact it is more than likely that excessive fluid plays a very important part in the compression ascribed to other factors, such as bone injuries, hemorrhages, etc., through interference with the return venous flow. Should there be a closing of the foramen of Magendie, for instance, distension of the ventricles producing hydrocephalus will occur. Again, should any local cause produce venous obstruction there will occur edema which in turn gives evidences of cerebral compression. I am inclined to think this plays a very important rôle as a causative factor in many obscure cases. Not infrequently, following injuries, the patient will remain conscious for a few hours with few if any symptoms. Then follow evidences of cortical irritation, as twitching and jerking movements, possibly headache, until the patient becomes exceedingly restless. This, in turn, is rapidly followed by stupor and finally coma. I believe these cases of acute compression must be due to this cause.

Diagnosis of compression has usually been made by three cardinal symptoms: headache, vomiting, and choked disc or papilledema. The term "choked disc" is an unfortunate one, since it depends upon the amount of edema present whether the ophthalmologist describes it as a choked disc or an optic neuritis. The term "papillitis" is objectionable because it implies an inflammatory reaction. Papilledema more clearly expresses the condition than any other. Following these cardinal symptoms we find mentioned: slow pulse rate, increased blood pressure, inequality of the pupils, changes in the reflexes, changes in the mentality, occasional convulsions, obstinate constipation and finally varying degrees of paralysis. The majority of writers state that if one of the three cardinal symptoms mentioned above be absent then the diagnosis of brain pressure must be gravely doubted. It is not so very long ago, in surgery of the gall-bladder, that men believed jaundice should be present before diagnosis of stone could be made. Now we realise, if we are going to operate these patients, that diagnosis ought to be made previously to the time of duct obstruction. Possibly the time may arrive when we will make our diagnosis of brain pressure equally early without waiting for these typical pictures to occur. Usually when a tumor of the brain has assumed proportions sufficient to produce these symptoms it at that time produces serious pressure, and operative measures must be instituted with the object of temporary relief rather than that of curing the patient. The time for radical extirpation in these cases is usually occupied in making a diagnosis and their hope in the future lies in early diagnosis, before the opportunity for radical operation is lost.

Take, for instance, papilledema. There is still considerable doubt how these lesions should be interpreted; but I presume we may have a papilledema separate and distinct from any optic degeneration. I can imagine a small tumor located posteriorly in such a way that it very early increases the fluid in the fourth ventricle, thereby producing papilledema. Here the eye symptoms would be among the very earliest signs, but in the majority of tumors, and other lesions of the brain producing pressure, papilledema must necessarily be one of the late signs. Autopsy records of cases of brain tumor show choked disc to be present in over 90 per cent. of the cases. This is not a fair way of interpreting the importance of this sign since when the case comes to autopsy the tumor has doubtless accumulated sufficient pressure to cause choked disc. If choked disc be present in a given case it is a very valuable and almost pathognomonic sign; but should it be absent one can not rule out the possibility of tumor. Furthermore, in the beginning of these cases of papilledema it is very difficult to state positively whether the condition be due to atrophy of the nerve, associated with nephritis or syphilis, or whether it be secondary to a tumor. Again, in experimental work I am told, where artificial pressure has been maintained over a considerable period of time, there are no changes in the disc. This makes it appear as if there were certain toxic elements associated with pressure in producing these lesions. On the other hand in certain cases where pressure has been relieved the improvement in the disc has been most marked.

Headache is of very great importance. It is almost always present and in many instances almost uncontrollable. In our cases it has been of no value in localising the lesion. The frontal tumors, as well as the cerebellar growths, have usually had headache in front as well as behind. In one reported instance of abscess in the frontal lobe the headaches were severe, but entirely occipital. In two of our cases there was a distinct history of headache previously; but when seen by us it had disappeared. The same might be said of vomiting. All our cases give histories of projectile vomiting, but in at least two instances it had entirely ceased.

Inequality of the pupils, if you can rule out any preëxisting lesions in the eye, is fairly pathognomonic of pressure inside the skull; but at the same time there are so many lesions that can produce this inequality it is of no value in locating them. Careful examination of all reflexes should be made. Each reflex includes a sensory impression, a center, and a motor response. The simplest example of this lies in the knee where, after tapping the patellar tendon, the impulse is carried up the sensory nerves and the posterior roots to the anterior horns in the lumbar segments, while from there the motor response is carried by means of the anterior roots and the motor nerves. If there be a lesion anywhere in this circuit there is absence of this reflex. Ordinarily, it is supposed, in cerebral lesions there is an exaggerated reflex because the inhibitory control is lost. It is positive there is some control above the primary arc because in complete transverse lesions of the cord above this point there is absence of reflexes. Cerebral lesions, then, usually give exaggerated reflexes.

The relation of the blood pressure to cerebral compression is most interesting. The work of Leonard Hill along these lines is as entertaining as any story book. "On one hand there is cerebral compression producing anemia, held in check on the other by the vasomotor appeal to the circulation for help. This help comes through increased arterial tension. As compression increases more urgent appeals are sent until from vasomotor fatigue the arterial pressure drops permanently below the pressure on the medulla. Respiration then fails and the uncontrolled heart finally allows arterial pressure to drop to zero."

Convulsions following cerebral injuries are quite frequent, but in only one type do they help us in any way in making a diagnosis. The presence of convulsions following injuries is a very definite sign of a brain lesion, but unless the convulsions be of the Jacksonian type it will not help toward locating the lesion. Convulsions having their expression in, say, the left hand, are a very definite and positive indication that the lesion is located in the right motor area. The same may be said of paralysis. When it occurs in a given part of the body it is positive that the lesion is located in the corresponding part of the motor area. This is, of course, exclusive of those lesions occurring in apoplexy which are located in the internal capsule. Spasm of any extremity may be present rather than complete paralysis. This is an evidence of cortical irritation on the opposite side, and it may be present in lesions of the base where hemorrhage has extended upward to the cortex. It is very important to ascertain if the paralysis came on immediately or if some minutes elapsed between the injury and the paralysis. If there have been any interval of time between the two, the paralysis is quite likely due to clot. What has been said of the motor area is equally true of any of the other brain centers.

The withdrawal of spinal fluid from the second lumbar space is often of the greatest help in diagnosis. It has been a wonder to me why this procedure has not been utilised more frequently when, by such a simple expedient, we are able to learn so much in certain brain and cord injuries. Because of the communication between the spinal and cerebral fluids the presence of any great quantity of blood in one will show up in the other. So in an injury of the skull where the diagnosis rests between hemorrhage and, we will say, concussion, if there be any quantity of hemorrhage present positive diagnosis can immediately be made by withdrawal of a small amount of spinal fluid. This, not infrequently, aids materially in relieving brain pressure under these circumstances. In at least one instance that occurred recently very marked improvement followed the withdrawal of the fluid. This is equally true of injuries to the spine because, where a clot is located in the upper portion of the cord, the spinal fluid below will be tinged with blood. There is one notable exception to this fact. Only very recently, in a crushing injury to the spine, I introduced a needle into the subdural space and was unable to withdraw any spinal fluid although I did get half a dozen drops of blood. At operation I found the spine crushed so as to completely sever the cord, and because of the opening in the dura the spinal fluid escaped into the cellular tissues. While this procedure is of very great importance in cases of injury to the head or spine, its use in compression due to new growths is fraught with considerable danger. There have now been about twenty deaths reported following lumbar punctures in tumors of the brain located posteriorly. While there has been frequent mention of this point in literature I am sure it needs further emphasis as there are still men who make this a routine practice in these cases. I am aware of the good results occasionally happening, like the relief of headache, etc., where it has been done with a true appreciation of its danger, but this is altogether different from using it as a routine method of diagnosis. All of the reported deaths have occurred inside of thirty-six hours, but many have died within a few minutes. Death is probably due to pressure on the respiratory center in the medulla.

The value of the microscopic examination of the spinal fluid, has been somewhat exaggerated. A differential count may prove of value while a positive bacteriological culture will be of the greatest importance in many cerebrospinal infections.

The diagnosis of pachymeningitis and leptomeningitis depends chiefly upon two things. First, convulsions. When these are present with a history of injury and begin in one part of the body, later becoming general—in other words, an epilepsy of the

Jacksonian type—it is exceedingly suggestive. If, however, it should be located in a portion of the brain remote from the motor area, then convulsions will probably be general in character. We must not forget that many so-called general convulsions may be of the Jacksonian type had we only sufficient knowledge of cerebral localisation to recognise them as such.

Second, limited areas of paralysis. These will differ according to the part of the dura involved. If the dura over the speech center, for instance, be involved we will have changes in speech varying from a hesitation to complete aphasia. So, as far as the extremities are concerned, these changes may vary from slight muscular weakness to complete paralysis depending upon the extent of the process. Occasionally we have a third factor in the complete change in the mentality of the individual as, for instance, in case one, we find him changed from a quiet, docile man to exactly the reverse. The Associated Press has recently given undue notice to such a case.

Localisation of brain tumors is an exceedingly difficult problem. The ground has been very thoroughly covered by a very large number of papers within the last few years. In fact, I believe that about as much has been accomplished by the neurologists and surgeons as is possible until the physiologists come to our relief by giving us a more accurate knowledge of cerebral localisation. Clinically, we try to divide tumors into those including the frontal lobe, the motor area, the cerebello-pontile angle and the cerebellum. At times incoördination, usually associated with cerebellar tumors, can be caused by frontal tumors. It is further believed when one cerebellar lobe becomes involved that its function is carried out by the opposite frontal. This at times makes diagnosis between the two well nigh impossible.

Each case should receive a very careful and thorough physical examination. In many cases there will be a complete absence of localising symptoms and only by most painstaking study are we able to recognise these when they are present. We should make a careful study of reflexes; paralysis; involvement of more especially the cranial nerves; ataxia and incoördination (both in the recumbent position and walking, with the eyes open and with the eyes closed); and then carefully consider to which part of the brain these point. Regarding the value of Babinski's adiodokokinesia sign I had not observed it in our cases. The accurate localisation of these growths, under the most favorable circumstances, is exceedingly difficult, while in a large percentage the almost complete absence of signs will make it well nigh impossible. At present the diagnosis in these cases is largely a matter of exclusion. Speaking very generally changes in mentality, cortical irritation, and the like, point toward the frontal lobes; involvement of the cranial nerves, especially the auditory, to the cerebello-pontile angle; while the general absence of symptoms with evidences of incoördination lead us to suspect the cerebellum. There are exceptions, but the rule is, when the symptoms are more marked on one side than on the other, the tumor will be on the corresponding side of the cerebellum.

In the treatment of cerebral injuries the ordinary trephine has almost passed into history. For exploratory purposes it gave us altogether too small an opening, and consequently it was essential to devise some means of exposing a larger area of brain. This is now best accomplished by means of the bone flap. By drilling the skull in several places and connecting these openings with any particular instrument one desires, either a cutting forceps or a Gigli saw, and then breaking the base of the flap, we can expose any given area of the brain desired. In this instance we have the added advantage of restoring the skull to its original contour as, when the operation is completed, this flap is replaced in its normal position. In exposing the brain by this flap method it is imperative that the periosteum be kept attached to the bone, otherwise necrosis will follow. This is best accomplished by a forceps (Krause's) made after the fashion of an ordinary volsellum, which holds skin, periosteum and bone in place.

Heretofore in these lesions we have been accustomed to treat the brain with too great deference. In epilepsy of the Jacksonian type I think it is advisable to completely excise the center rather than simply separate any adhesions which happen to be present. Paralysis will of course follow for a certain length of time, but in at least two instances that I recall, there has been complete recovery following this method. These patients have now been well for three years. The same may be said of cases of local pachy- and lepto-meningitis as it is not sufficient to merely expose without complete excision.

As a general principle when pressure is present it must be relieved. Where the condition that produced the pressure is accessible it should be removed; but if it be inaccessible, either through location or great size, we can accomplish a similar purpose by excising a certain amount of the skull, thereby giving the brain an opportunity to occupy more space.

Fortunately, while we cannot accurately localise the tumors, we can diagnosis compression and can alleviate several of its most distressing symptoms. For instance, headache is often continuous, total loss of vision a certainty; while in decompression we have a therapeutic measure which usually gives temporary and occasionally permanent relief. Leaving aside those cases apparently cured by decompression the temporary relief afforded many others is ample justification for its more general use. Regarding the location, the right subtemporal opening is most popular. This, of course, should only be true where the speech center is located on the left side; in left-handed people it should be made on the left side, because here the speech center will probably be on the right side.

Regarding the use of decompression in cases of fracture of the base we have had scarcely any experience. In view of the experiments carried out by Crile it is certain that many of these cases of fracture of the base are literally dead, although they continue to breathe and their pulse still beats. In other words the pressure is sufficiently great to destroy the centers and no amount of decompression under these circumstances will avail. I do not doubt that in the less serious cases decompression may be of material benefit. It would seem as though we ought to go slowly in advising this operation in these cases. In the first place probably the majority of fractures of the base get well under the expectant plan of treatment. Quite a number of serious ones will die no matter what be done, because the pressure has lasted sufficiently long to destroy the center. Therefore it leaves but a small number of cases where it would be advisable to employ decompression. However, it has always seemed to me we have been content to allow our hands to be tied in these cases. I know, in times past, I have allowed cases to die without any attempt at release of pressure where a decompression might have been of benefit. I thoroughly bel'eve that in a certain proportion of the serious cases decompression is a step in the right direction; and in the not far distant future we shall probably resort to even more radical measures, to save these cases.

Decompression on both sides has met with little favor as there seems to be a greater mortality in this operation than when it is done on one side only. I know of no operation requiring the use of greater judgment than decompression. For instance, if the opening be made too large, and there be great pressure present, a hernia will insue probably causing motor paralysis. The question arises: How can this be obviated? In the subtemporal operation this is less likely; but even here I have known it to occur. It would seem to be wise where there is any great tension to merely remove the bone without opening the dura. If, after several days, the tension remain great make a decompression on the other side without opening the dura; then, when the pressure has been equalised, open the dura on the right side. It is just possible that in this way some of the untoward accidents may be avoided.

Regarding the subtentorial decompression I have had very little experience. It would seem to me it were a matter for exceedingly careful consideration to decide upon an exploration or a decompression; and, if a decompression be decided upon, whether it should be subtentorial or subtemporal.

In cases of chronic meningitis involving either the dura or the pia, where the condition is local, these areas ought to be excised. These lesions are very frequently tubercular and not infrequently it is impossible, on account of the extent of the condition, to completely excise them. Here we have a condition very similar to that of tuberculosis of the peritoneum: in each case a serous membrane is involved and if, in case of the peritoneum, simple exploratory operation frequently results in a cure it would seem that in the other instance exploratory operation would prove of similar benefit. So in tubercular lesions of the meninges, if complete excisions be impossible it is certainly wise to expose them to the air, puncturing any small collections of fluid which may be present.

Up to the present time there has been very little success in the treatment of the general infections of the meninges; but if we look back on a similar condition of the peritoneum it is only a few years ago that we considered it to be practically hopeless. Now we know it is largely a question of the time that has elapsed between the perforation and the operation as nearly all the early cases get well. Satisfactory drainage here is very difficult, but if we could open our cases of general infection of the meninges early, and could establish free drainage doubtless much improvement would follow.

Irrigation here is as much under discussion as it is in the peritoneum. Personally, I do not believe in irrigating the peritoneum, but as yet my experience in the cranium has been too limited to be of any value.

Regarding the use of urotropin in these cases we have not met with the success anticipated. From the experimental work, however, I believe it should be given a very thorough trial. Very likely its chief use will be in the prevention of infection rather than in the treatment once it is established. I believe for the present that in all injuries to the skull, where there is a possibility of infection, this drug should be used. I should further advise its use preparatory to all operations upon the central nervous system.

Case I. Pachymeningitis. Some months before patient fell out of a wagon striking his head but was not aware of any injury at the time. Ten days later, however, he was seized with convulsions. These at first occurred about once a week and later more frequently. Prior to the accident he was gentle, kind and docile. Afterward there was a distinct change in the mentality of the patient and he developed fits of temper, wanting to fight with people and later would remember nothing about it. The convulsions were not of the Jacksonian type. Discs were reported unusual; blood pressure, 110. On inspection, after having shaved his head, a certain deformity of the skull was seen which was evidently the seat of injury. At operation, by Dr. Roswell Park, a bone flap was raised over this area which exposed a pachymeningitis quite limited in extent. There were a number of areas which, on puncturing with a needle, contained fluid, not unlike a localised edema; and scattered between these were several whitish streaks suggestive of tuberculosis. The dura over this area was completely excised, the bone flap replaced, and up to the present time, now about two years after the injury, there has been no return of the convulsions and his mental condition has markedly improved. A recent communication says he is now engaged in managing a large business with complete restoration of his health. There has been no return of convulsions.

Case II. Leptomeningitis. J. S. entered the wards of the Buffalo General Hospital giving a history of a fall some months before. He first noticed a difficulty in moving his right leg. Later the arm became involved. Finally almost complete paralysis of the speech center ensued. He was able to articulate to the extent of saying "yes and "no," but nothing more. This case showed no evidences of brain pressure. Reflexes were very markedly exaggerated. No change was present in discs, and blood pressure was 115. Operation showed a number of areas similar to those found in Case No. 1, only they were located in the pia-arachnoid instead of the dura. Cultures taken from the puncture fluid proved negative and, on account of the extent of the lesion in this particular case, I was loath to completely excise the pia. After two or three days following operation, he was able to say a few more words; and about six weeks after the operation, he could run two or three words together. The spasm of the right arm and leg has almost entirely disappeared. The reflexes are still exaggerated but not to the same extent as before.

Both cases illustrate first, the value of exploration in these doubtful cases; and second, the possibilities of surgery in local pachy- and lepto-meningitis.

Case III. As this case presents some rather peculiar conditions I will record it more in detail. His family history is negative with the exception of one sister dying of tuberculosis and one sister now ill with some form of insanity. The patient was 45 years of age and a contractor by occupation. His health had always been of the best. He had engaged in outdoor work all his life, and said that up to his present trouble he had never been ill in any way. His present illness began about three months previous to the time of consulting us, with a buzzing in his right ear. This gave him no particular trouble, but as it continued he consulted a doctor at his home in Vancouver, B. C. During the examination, which included the eyes and ears, an edema of the discs was discovered. Nothing abnormal was found with the ears. About the same time he began to have headaches. These were for a time of marked severity. Occasionally at this time he had projectile vomiting. After about a month the headaches grew very much better and the vomiting ceased. The disturbance of the ear practically subsided and were it not for the fact that examination of his eye showed a progressive papillitis he would have been regarded well. Patient said that he noticed some twenty-six years ago a difference in vision. On reading with one eye closed he found that the sight in the left was better than that in the right.

Patient had lost twenty pounds in weight, but said he had gained five or six pounds in the last two weeks. Examination at this time showed urine entirely negative, with an unusually high elimination. There was no lymphatic involvement nor other evidences of syphilis. Wasserman reactions, three in number, were all negative. Von Pirquet was negative. The blood showed 98 per cent. hemoglobin; 5,100,000 reds; leukocytes, 6; polynuclears, 75; small lymphocytes, 18; large lymphocytes, 6; eosinophiles, 1. Nothing abnormal noted in the character of the red cell. Blood pressure, 115.

Repeated eye examinations showed a double papillitis with hemorrhages. These progressed rapidly and it became evident that his sight would soon be gone. There was no change in the color fields; no nystagmus and no paralysis of the muscles; pupils even; reflexes were normal; ankle clonus and Babinski absent. There was no evidence of ataxia. Patient walked equally well with eyes open and closed. There was no swaying with eyes closed and coördination was seemingly perfect. Adiodokokinesia sign absent.

Repeated examination of the ears failed to reveal any disturbance therein. There was no evidence of an involvement of any of the cranial nerves nor of either motor or sensory paralysis. There were no convulsions. In fact in summing up the man presented slight headache, more or less constant, and a rapidly increasing papilledema with marked hemorrhages into the discs.

The eye examinations were made by Dr. Crosby of Vancouver, and Drs. Howe and Francis of Buffalo. The neurological side of the case was studied by Dr. Putnam. In view of the rapidly increasing papilledema, the continuous headache, and the absence of localising symptoms, a decompression was advised. A subtemporal opening was made on both sides and the dura pulsated normally. The right dura was then opened and as no great tension was observed, the same was done on the left side. Here the pressure was greater, but still not particularly marked. Following the operation patient complained of being exceedingly lame and tired, but otherwise in good condition. In about six to eight hours, however, he became restless, throwing his arms and legs about the bed. This continued for about twenty-four hours when he was again taken to clinic, on the suspicion that some cortical irritation due to hemorrhage might be present. The flaps were reopened and it was discovered there was marked protrusion of the brain through both openings, but much more marked on the left than on the right. Nothing more was done in a surgical way. After returning to his room the patient rapidly grew worse, finally lapsing into deep coma.

Autopsy showed a marked protrusion of the brain through the left decompression opening; less so on the right. There was no fluid present, ventricles being apparently empty. Careful sections of the cerebrum failed to discover anything abnormal. In the cerebellum, however, on the left side was a distinct cavity, about the size of an olive, filled with old clot. Upon opening the left lateral sinus a clot was found which was evidently recent. Section failed to reveal any evidences of tumor formation in the cerebellum. Sections of the optic nerve showed many areas of hemorrhage.

Reviewing this case there are many points of interest. What produced the hemorrhage in the cerebellum and in the optic nerves? Was there any relation between the lateral thrombosis and the operation? A kidney lesion or syphilis might explain the hemorrhage, but this seems impossible in view of the facts: first, that three months' careful study of the urine failed to reveal anything abnormal; and secondly, repeated Wasserman reactions, both before the operation and after death, were negative.

I have very little doubt the evidences of acute compression following operation were due to thrombosis of the lateral sinus, but it is difficult to explain a thrombosis in the lateral sinus in so simple a procedure as a decompression. It is just possible the same unknown factor which produced the hemorrhage in the cerebellum was the cause of the lateral thrombosis. In any event this case illustrates how a serious cerebral compression may be due to interference with the return venous flow.

430 DELAWARE AVENUE.

